# Using the Novel “3D-Ortho” Concept to Achieve a Beautiful Asian Face

**DOI:** 10.7759/cureus.38535

**Published:** 2023-05-04

**Authors:** Koji Yamamoto

**Affiliations:** 1 Dentistry, Cozy Yamamoto Dental Clinic, Ama, JPN

**Keywords:** facial cosmetics, inclination, nasolabial angle, e-line, buccal corridor, orthodontics, extraction, maxillary skeletal expander

## Abstract

It has become more common for adult patients to seek conflicting orthodontic results, such as the reduction of the buccal corridors by arch form expansion and achieving esthetic-line (E-line) harmony by moving the anterior teeth backward using the extraction space. Therefore, the author devised and implemented the novel “3D-Ortho” concept to achieve the abovementioned conflicting results and solve the requirement of facial beauty. The patient was a 23-year-old female with mouth protrusion. The author used the 3D-Ortho concept to provide orthodontic treatment. A maxillary skeletal expander appliance and the extraction of the maxillary first premolar were performed, and a multi-bracket device was placed on the buccal aspect to decrease Mx1 to point A-pogonion line (APo) by full class II molar relationship finishing. Postoperatively, the nasolabial angle was increased from 83.4° to 93.1°, the E-line to upper lip distance decreased from 2.6 mm to −1.1 mm, the Mx1 to Apo distance decreased from 13.6 mm to 7.5 mm, the distance between the mesial buccal cusp of the mandibular first molars increased from 41.2 mm to 45.2 mm, the inclination of the mandibular first molar incisor changed from 21° to 11° on the right side and from 21° to 9° on the left side, and the improvement of the buccal corridors was observed; thus, significant improvement was observed in these parameters. Therefore, the novel orthodontic treatment method showed good results in solving problems unique to Asians, incorporating occlusal stability and facial beauty harmony.

## Introduction

Standard edgewise orthodontic treatment began with Angle’s non-extraction orthodontics and evolved with Tweed’s extraction orthodontic theory, followed by Andrews’ straight-wire orthodontic treatment and the development of no-friction bracket devices. As orthodontists focus mainly on tooth movement patterns, the advent of temporary anchorage devices (TADs) has facilitated complex tooth movements such as intrusion and distal movement, expanding the range of indications [1]. With the development of lingual orthodontic treatment, it became possible to also consider the aesthetic needs of patients, and, with the evolution of Invisalign, treatment with removable clear aligner orthodontic appliances has proven useful. The development of maxillary skeletal expander (MSE) appliances has enabled the lateral expansion of the maxilla by releasing the median palatal suture in adults, which was previously considered difficult [2]. Aesthetic factors, such as facial convexity, nasolabial angle (mouth protrusion), esthetic-line (E-line), buccal corridor, and the length of the lower face must be considered when patients are concerned with improving their appearance. However, it is difficult to majorly change the height of the lower face by orthodontic treatment alone because of the limited movement of the mandibular joint. Therefore, the author aims to create the illusion of a shorter lower face by changing its aspect ratio. The method involves expanding the arch form of the maxilla to increase its horizontal width and shortening its vertical length by counterclockwise rotation. With the increasing aesthetic demands of adult patients, they sometimes seek conflicting results. For example, the reduction of the buccal corridors is accomplished by arch form expansion, and E-line harmony is achieved by moving the anterior teeth backward using the extraction space. However, these two actions produce conflicting results, which have been considered a difficult task in the past. Therefore, the author devised a new treatment concept, “3D-Ortho,” to achieve two conflicting results and to solve the important requirement of facial beauty, which obtained good results, as reported here.

## Case presentation

**Figure 1:**
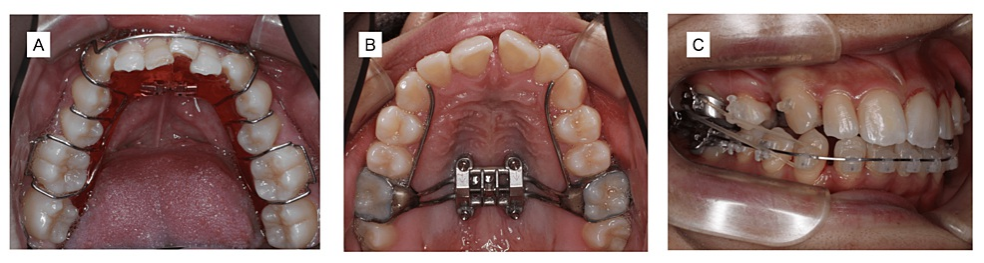
Photographs of the oral appliance (A) Mandibular views during lateral uprighting. (B) Maxilla views of enlargement of the arch form using an MSE appliance. (C) Distal uprighting of the mandibular molars using an MSE appliance with class Ⅲ elastics.

The proposed 3D-Ortho concept is an orthodontic method that combines the disadvantages of a narrow arch form by four-tooth-extraction orthodontics and the improvement of the E-line, which is difficult to achieve with non-extraction orthodontic treatment. The first step is to gain space in the dentition. The enlargement of the arch form is achieved by the lateral uprighting of the mandibular dental arch (Figure 1A), which is associated with the enlargement of the maxilla using MSE appliances (Figure 1B).

The second step is to decrease the incisor mandibular plane angle (IMPA), which is accomplished by the distal uprighting of the mandibular molars using an MSE appliance with class Ⅲ elastics (Figure 1C) or by inserting a TAD in the mandibular distal of the second molar portion. IMPA is decreased via an inter-proximal reduction of the anterior teeth. The third step is to shorten the lower face and decrease Mx1 to APo. In the case of angle class Ⅰ or Ⅱ, the maxillary left and right lateral premolar teeth are extracted to decrease Mx1 to APo by finishing the full class II molar relationship (Figure 1C).

**Figure 2:**
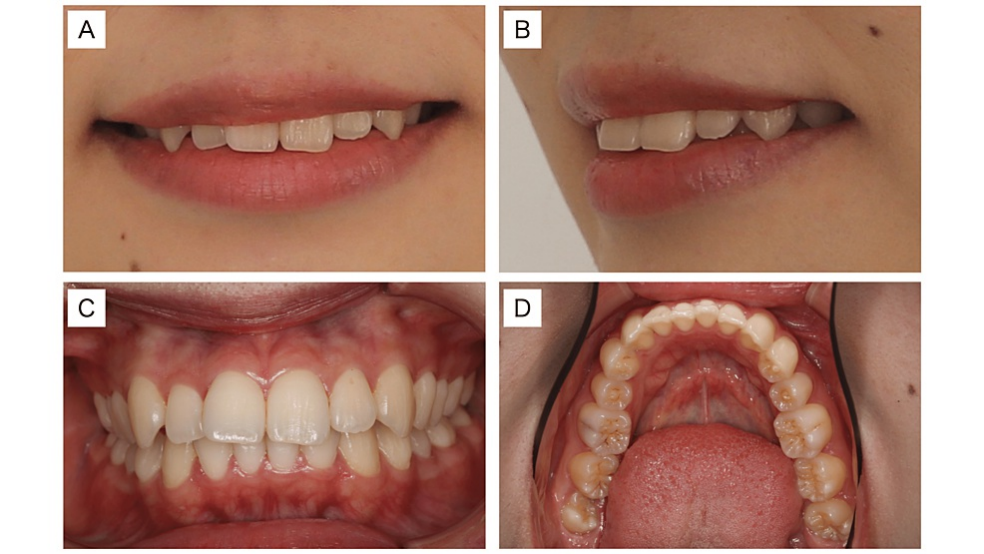
Preoperative facial and intra-oral views. (A) Smile and buccal corridor. (B) Three-quarter smile. (C) Intra-oral frontal view. (D) Intra-oral mandibular occlusal view.

Herein, the author reports the case of a 23-year-old Japanese woman with a chief complaint of protruding sensation around the mouth. She was physically healthy, and there was nothing special to note in her medical history or current illness. Her facial appearance showed a buccal corridor (Figure 2A, 2B) during her preoperative smile.

The CephaloMetrics AtoZ software program (YASUNAGA Computer Systems Co., Inc., Fukui, Japan) was used to analyze lateral cephalometric radiographs after the anatomic landmarks were marked. Her nasolabial angle was 83.4°, which was narrower than the average angle of 102.0° [3]. Her subnasale-pogonion line to upper lip was 9.0 mm, which was more progressive than the average distance of 6.0 mm, and her E-line to upper lip was +2.6 mm, which was more progressive than the average of 0.0 mm [4]. Intra-oral findings (Figure 2C, 2D) showed that the left side of the cuspids and molars was class I, whereas the right side was class II. According to Thompson’s classification, the maxillary and mandibular dental arches were round V-shaped. There were no temporomandibular joint sounds on the left or right side. The third molars were on the left and right sides of the mandible. The second premolar on the left side of the mandible showed apical periodontitis. Model analysis showed an anterior ratio of 71.2% and an overall ratio of 88.9%, with a particularly small anterior ratio. The total crown width was 112.3 mm in the maxilla and 103.8 mm in the mandible. The distance between the mesial buccal cusp of the first mandibular molars was 41.2 mm. Lateral cephalometric analysis indicated skeletal class I, with an SNA of 79.5°, an SNB of 76.9°, and an ANB of 2.6° (Table 1).

**Table 1:** Cephalometric skeletal analysis

Measures	Mean±SD	Pre-treatment	Post-treatment
SNA (°)	80.0±3.0	79.5	79.3
SNB (°)	77.0±3.0	76.9	76.9
ANB (°)	3.0±2.0	2.6	2.4
Facial angle (°)	87.8±3.6	87.7	87.8
Y-axis (°)	59.4±3.8	57.6	57.9
FMA (°)	25.0±6.0	17.2	17.7
L1-MP (IMPA) (°)	90.0±6.0	118.7	104.9
Mx1 to APo (mm)	2.7±1.8	13.6	7.5
Md1 to APo (mm)	3.4±2.0	8.6	4.9
Over jet (mm)	3.2±2.5	5.1	2.1
Over bite (mm)	2.3±2.0	2.2	1.5
E-line-upper lip (mm)	0.0±2.0	2.6	-1.1
E-line-lower lip (mm)	2.0±2.0	3.5	-0.3
Nasolabial angle (°)	102.0±8.0	83.4	93.1
Sn-Pog to upper lip (mm)	6.0	9.0	5.1

Vertically, the FMA was 17.2°, and the branchy-facial pattern was smaller than the standard deviation (Table 1).

In terms of the patient’s dentition, Mx1 to APo and IMPA were 13.6 mm and 118.7°, respectively, which were larger than the standard deviation. The long axis of each first molar was determined, and the inclination of each molar was measured using the long axis and the floor. Cone-beam computed tomography (CBCT) findings showed that the mandibular first molar had a lingual inclination of 21° on the right side and 21° on the left side, which was greater than the standard deviation. The diagnosis was a skeletal class I, right angle class II with anterior crowding. The anterior positions of the upper and lower lips needed to be improved to achieve a harmonious facial appearance. Considering the E-line, the author decided to project the mandibular anterior teeth from the current Md1 to APo of 8.6 mm to 4.9 mm, a decrease of 3.7 mm, according to the visual treatment plan.

To avoid the narrowing of the arch form in the four-tooth-extraction orthodontic treatment, the mandible was enlarged without extraction based on the 3D-Ortho concept, the maxilla was enlarged by MSE appliances, and the maxillary first premolar was extracted to achieve close occlusion with full class II finishing. MSE appliances (four fixed screws: 9 mm in length, 1.5 mm in diameter, forest-one) were placed in the maxillary palate, and the screws were implanted. At the same time, a holding arch was placed in the mandible, and lateral uprighting of the mandible was started. Next, extraction of the maxillary first premolar was performed. A multi-bracket device was placed on the buccal aspect, and leveling was started using 0.012” NiTi wire. After the treatment was started, the wires were changed to 0.012” NiTi wire, 0.014” NiTi wire, 0.016” NiTi wire, and so on. Torquing was done with 0.016” x 0.022” NiTi wire and 0.019” x 0.025” NiTi wire, and detailing was done with upper- and lower-jaw 0.016” x 0.016” TMA wire to tighten the occlusion.

**Figure 3:**
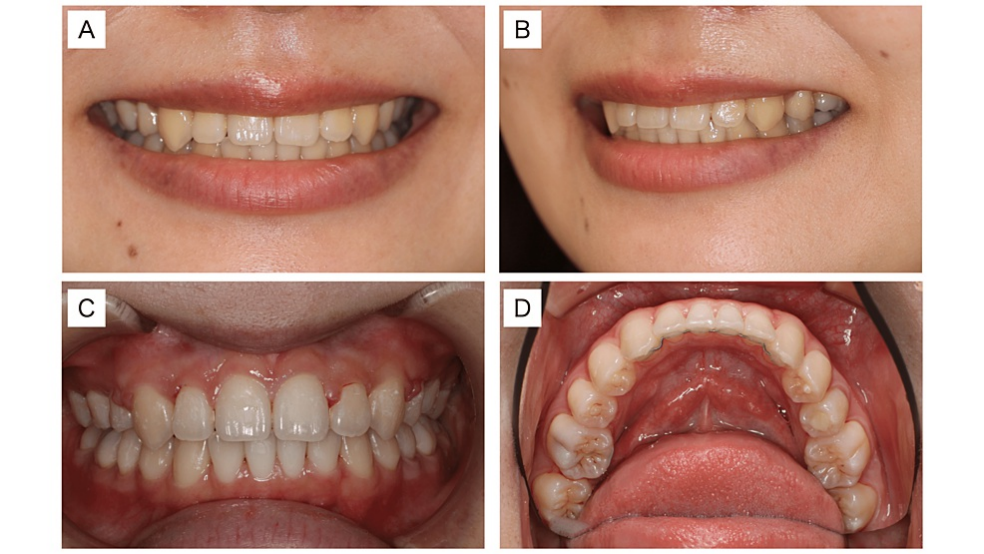
Postoperative facial and intra-oral views (A) Smile and buccal corridor. (B) Three-quarter smile. (C) Intra-oral frontal view. (D) Intra-oral mandibular occlusal view.

In terms of facial appearance, the buccal corridor (Figure 3A, 3B) seen in the preoperative smile was improved, and the smile line was changed so that it was in harmony with the lip line.

**Figure 4:**
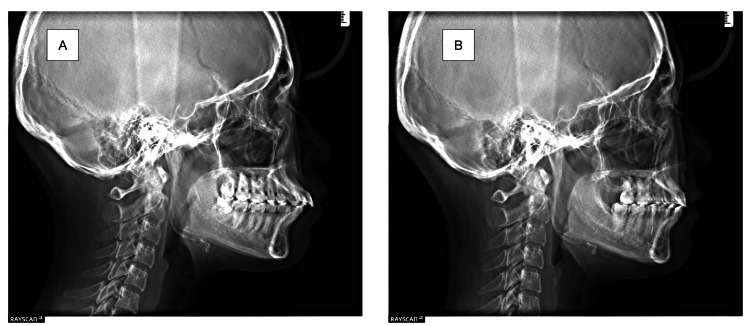
Comparison of lateral cephalometric radiographs (A) Preoperative lateral cephalometric radiograph. (B) Postoperative lateral cephalometric radiograph.

**Figure 5:**
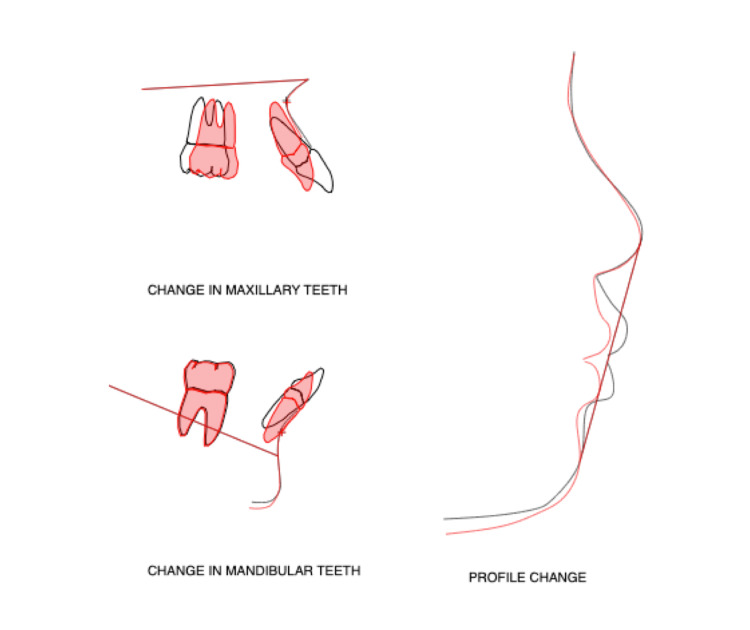
Profile changes post-treatment Image credit: Koji Yamamoto

The nasolabial angle, subnasale-pogonion line to upper lip, and E-line to upper lip were improved significantly, which were changed from 83.4° to 93.1°, from 9.0 mm to 5.1 mm, and from 2.6 mm to −1.1 mm, respectively. In the intra-oral (Figure 3C, 3D), the maxillary and mandibular dental arch was enlarged, resulting in a U-shaped dental arch. Thus, a single-tooth-to-two-tooth cusp fit and functional occlusion of the lateral teeth were obtained. Upon comparing the initial examination and the results at the end of dynamic orthodontic treatment, a lateral cephalometric analysis showed that the SNA and SNB increased slightly from 79.5° to 79.3° and from 76.9° to 76.9°, respectively, and the y-axis changed only slightly from 57.6° to 57.9° (Figures 4, 5; Table 1).

**Figure 6:**
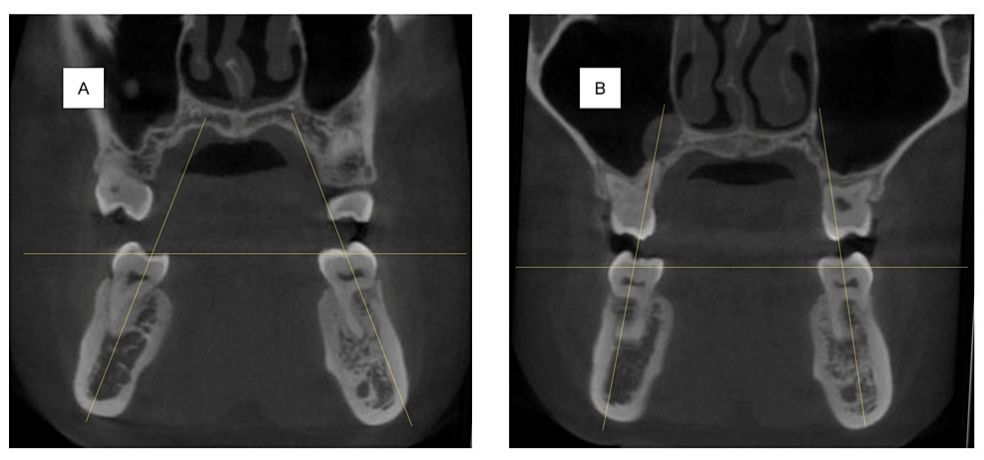
Coronal view of CBCT scan showing angular measurements of mandibular first molars (A) Preoperative CBCT image showing the coronal section of mandibular first molars. (B) Postoperative CBCT image showing the coronal section of mandibular first molars.

Furthermore, there was almost no mandibular rotation. In the tooth system, Mx1 to APo and Md1 to APo decreased significantly after treatment from 13.6 mm to 7.5 mm and from 8.6 mm to 4.9 mm, respectively. In the model analysis, the distance between the mesial buccal cusp of the mandibular first molars increased from 41.2 mm to 45.2 mm after the dynamic orthodontic treatment. CBCT showed that the inclination of the mandibular first molar decreased from 21° to 11° on the right side and from 21° to 9° on the left side (Figure 6; Tables 1, 2).

**Table 2:** Indications between the mesial buccal cusp of the mandibular first molar distance (mm). The inclination degree of the mandibular first molars (i.e., the long axis of each first molar) was determined, where the inclination of each molar was measured using the long axis and the floor

	Pre-treatment	Post-treatment
Between the mesial buccal cusp of the mandibular first molars distance (mm)	41.2	45.2
The inclination of mandibular right first molar degree (°)	21	11
The inclination of mandibular left first molar degree (°)	21	9

## Discussion

As the initial stage of cosmetic surgery for facial beauty, the need for adult orthodontic treatment has increased markedly, and patients are no longer satisfied with just having beautifully aligned teeth. The three-dimensionality of the midface, which is an important element of facial beauty, is greatly influenced by the position of the subnasal point, as reported by the author [5,6], which significantly influences the nasolabial angle. There are many Asians who have a bimaxillary protrusion facial profile, lips that surpass the E-line, and narrow nasolabial angle. A narrow nasolabial angle is associated with lip protrusion [7], such that the lips appear to be protruding even if the E-line is well-defined. In order to correct the alignment of the teeth and the position of the anterior teeth, bone expansion using MSE appliances, distal uprighting of mandibular molars, inter-proximal reduction of anterior teeth, forward movement of anterior teeth, and extraction of teeth are used to obtain space.

In recent years, there have been reports of the use of MSE appliances (e.g., rapid expansion devices using orthodontic anchor screws as a fixed source) to release the median palatal suture. Although periodontal tissues often need to be considered when orthodontic treatment is performed on adults, incorporating jawbone expansion into treatment may be very effective in this case, as it reduces tooth movement compared to orthodontic extraction. In addition, because Caucasians have a more anteriorly sculpted midface compared to Asians [8-10], typically, their E-line is originally well-defined, their dental arch width system is narrower than that of Asians, and maxillary expansion via MSE appliances is very useful for space acquisition. However, as the facial features of Asians include protruding lips and an underdeveloped chin and tip of the nose, extraction of teeth is often necessary when considering lateral facial aesthetics based on E-line. Extraction orthodontics tends to cause increased buccal corridor, increased nasolabial folds [11], concavity of the buccal area, and increased lower facial height [12]. When MSE appliances are used to enlarge the jawbone and gain space, the arch form is not narrowed as in extraction orthodontics, but it is difficult to significantly improve the protrusion of the mouth.

According to Dawson, there are two reasons for the existence of the curve of Wilson [13]. First, Dawson observed an optimal resistance to loading, whereby the buccolingual inclination of the posterior teeth parallels the inward pull and orientation of the internal pterygoid muscle contraction to produce the greatest resistance to masticatory forces. Second, the inward inclination of the occlusal table allows open access to food as it is being chewed, facilitating the masticatory process. The lingual inclination of the mandibular molar is significant in both anatomy and function. As described by Dawson, the curve of Wilson is essential for anatomical stability as well as masticatory function. Dawson also stated that when the curve of Wilson is made too flat, ease of masticatory function may be impaired. In the literature, the buccolingual inclination of the mandibular molars ranges from 12.59° to 15.7° [14-17]. In this case, the right side of the mandibular first molar’s inclination was 21° preoperatively and 11° postoperatively, and the left side was 21° preoperatively and 9° postoperatively. Furthermore, buccal uprighting was used to obtain a more stable occlusion.

## Conclusions

This work showed that the 3D-ortho concept can achieve an attractive buccal corridor and an appropriate E-line setting from the viewpoint of facial aesthetics, as well as appropriate inclinations of the mandibular first molars in terms of occlusion. In this paper, the author reports a novel concept of orthodontic treatment that incorporates a cosmetic element based on problems unique to Asians, and favorable results were obtained with a highly safe method. In the future, the author plans to increase the number of cases and further examine the stability of the postoperative retention period.
